# Contextualizing Contemporary Research Ethics Policies and Practices in Significant Historical Events

**DOI:** 10.1007/s40617-023-00865-2

**Published:** 2023-11-02

**Authors:** Sarah C. Mead Jasperse, Michelle P. Kelly

**Affiliations:** Emirates College for Advanced Education, c/o Emirates College for Advanced Education, Khalifa City A SE-43, Abu Dhabi, United Arab Emirates

**Keywords:** Research ethics, Applied behavior analysis, Policy, Legislation, Code

## Abstract

The *Ethics Code for Behavior Analysts* (BACB Code; Behavior Analyst Certification Board®, 2020) includes six items in “Section 6—Responsibility in Research'' that cover the protection of human participants in research activities conducted by Board Certified Assistant Behavior Analysts® and Board Certified Behavior Analysts®. This article provides a brief primer regarding significant historical events and foundational documents that have resulted in the contemporary research ethics policies and practices in the field of behavior analysis. This walk through the last century from the Nuremberg Code to the Declaration of Helsinki, the Belmont Report, and 45 CFR Part 46—Protection of Human Subjects provides a summary of why the codification of requirements such as “informed consent” and “Institutional Review Board” oversight was necessary. The linkages between these historical events and the current BACB Code items are discussed. In addition, situations in which the BACB Code does not provide explicit instruction but foundational documents may provide additional guidance are considered. Finally, opportunities for future data-driven decision making in research ethics are offered.

The Behavior Analyst Certification Board’s *Ethics Code for Behavior Analysts* (hereafter referred to as the BACB Code) reaffirms what has been a long-standing pillar in the field of behavior analysis: All clinical and treatment activities must be based on research findings (Behavior Analyst Certification Board [BACB], 2020). Section 2.01 states that behavior analysts “provide services that are conceptually consistent with behavioral principles, based on scientific evidence, and designed to maximize desired outcomes for and protect all clients, stakeholders, supervisees, trainees, and research participants from harm” (BACB, 2020, p.10). This positions research as a critical activity within the field; if there is no base of scientific evidence, then there is no clinical work.

Given the essential nature of research, the BACB Code also includes a section dedicated entirely to ethics standards related to research: Section 6—Responsibility in Research. This section includes 11 items that are designed to guide research activities conducted by Board Certified Assistant Behavior Analysts® (BCaBAs) and Board Certified Behavior Analysts® (BCBAs). The first six items specifically address the protection of human participants in behavior analytic research. As is standard in a professional code, these items are written as straightforward instructions without any explicit reference to the historical variables responsible for the development of the code items. However, behavior analytic researchers may benefit from a contextualization of contemporary research ethics policies and practices in the significant historical events and foundational documents that played a role in the creation of modern standards.

The benefit may be twofold: (1) Behavior analytic researchers may better understand the *why* behind the *what* of the BACB Code and take into account the devastating consequences that could result from not adhering to the BACB Code items; and (2) When faced with novel research ethics situations, if behavior analytic researchers are familiar with the historical context and foundational research ethics documents upon which the BACB Code is built, then the researchers may be able to respond safely and effectively to circumstances for which the BACB Code does not provide direct guidance. Likewise, behavior analysts who are not certified by the BACB and, therefore, are not bound by the BACB Code may find value in reflecting on the same historical variables when considering their own practice. For example, if behavior analysts are certified by other bodies (e.g., Qualified Applied Behavior Analysis Credentialing Board [QABA]®), they may find that the relevant ethical standards (e.g., *QABA Ethical Code of Conduct*; QABA, 2021) link to the same foundational research ethics documents. This contextualization may be particularly timely as practitioners currently contribute substantially to the research that is published in peer-reviewed journals in the field of applied behavior analysis (ABA). Since 2017, nearly half of the research articles published in the *Journal of Applied Behavior Analysis* (JABA) have included at least one author with a listed clinical affiliation (Normand & Donohue, 2023, p. 14). In addition, behavior analysts who are practicing outside the United States of America (USA) and are developing their own national professional standards, including items related to ethical practice, may find utility in returning to significant historical events and foundational research ethics documents during the task of drafting the standards (for a full discussion regarding the development of national occupational standards for behavior analysis, see Kelly & Trifyllis, 2022).

Thus, the purposes of the current article are to:

- Provide a brief primer of significant historical events and foundational research ethics documents relevant to contemporary research ethics practices in the field of behavior analysis.
- Identify links between these significant historical events and foundational research ethics documents and the items in Section 6 of the BACB Code.
- Offer example scenarios in which the BACB Code (or alternative behavior analytic professional codes) might not give explicit guidance to behavior analytic researchers but foundational research ethics documents may provide direction.
- Suggest ways in which behavior analytic researchers might make data-driven decisions about research ethics in alignment with the BACB Code and foundational documents.

## A Brief Primer and Historical Timeline of Significant Events and Foundational Documents in the Area of Research Ethics

What follows is a brief primer about some significant historical events and foundational documents that have been critical to the development of contemporary research ethics standards. This primer is not meant to tell the full story of all events nor can it ever replace what can be learned by reviewing the primary sources directly. Rather, this primer is meant to reduce response effort by conveniently bringing together and summarizing information that has been published in disparate locales, to provide the reader with a robust reference list that can be used as a starting point to dig deeper into each of the events and documents described, and to provide the base upon which links to the field of behavior analysis can be made in the subsequent section.

### Early Behavioral and Medical Research with Human Participants (1920–1939)

Although questions about how the human body functions and why people behave the way they do probably have been around as long as humans have existed, this primer begins in the early 1920s to emphasize research activities that have occurred under reasonably similar circumstances to today’s research activities (e.g., often in a university or clinical setting, to answer specific research questions, with the purpose of disseminating the findings to change practice or policy). The three research projects briefly summarized here were selected to represent the types of research activities that occurred with some degree of regularity before modern policies were established. Of course, the summaries do not do justice to the complete and complex stories of those who suffered as a result of these research projects. Readers are encouraged to consult cited sources for further details.

#### Little Albert Research (1920)

The story of Little Albert is frequently told in introductory psychology textbooks—both as a cautionary tale and to share the empirical findings (Harris, 1979). In 1920, John Watson and Rosalie Rayner described a research project in which they conditioned a fear response to be demonstrated by an infant, Albert, in response to a previously neutral stimulus (a white rat) by pairing the stimulus with the loud sound of a hammer hitting a steel bar. Through various follow-up analyses to test generalization and further conditioning, Albert’s fear response was subsequently conditioned to other stimuli and explicit steps were never taken to reverse the conditioning (Harris, 1979). Many uncertainties and mysteries surround the Little Albert research activities (for further discussion on the topic, see Digdon, 2020; Digdon et al., 2014; Frilund et al., 2020; Harris, 2020). Some of these claims include suggestions that Albert might have been born with a neurological impairment (Frilund et al., 2020) and that Watson and Rayner knew that Albert was going to be discharged from the hospital before the conditioning could be reversed (Harris, 1979). Establishing the veracity of these claims is far beyond the scope of the article, but, nonetheless, the Little Albert story is a cautionary tale about the responsibility a researcher must take if working with a vulnerable participant and studying a behavioral phenomenon that both could be harmful to the individual and serves no direct benefit for that person.

#### Tuskegee Syphilis Study (1932–1973)

In 1932, a group of medical researchers began studying syphilis at the Tuskegee Institute in Alabama, USA (Tobin, 2022). Hundreds of Black men with syphilis, many of whom were economically and socially vulnerable and unaware of their diagnosis, were enrolled in the study under the illusion that they would be receiving free medical care. Instead, the researchers were interested in learning about untreated syphilis and willfully withheld known treatment regimens. Even after many of the participants died, the study continued until information about the project made its way into the mainstream news in a *Washington Star* article in 1972 (Tobin, 2022). The 40-year study was subsequently halted and discussed at legislative hearings that set the stage for the development of the National Research Act of 1974 (discussed later in the current primer).

The abhorrent treatment of the men in this study is a sobering reminder that deception in research can lead to life-and-death stakes, especially when working with vulnerable populations. Furthermore, this is an example of one group bearing an undue burden in research. The participants in this project were subjected to extreme risk whereas the benefits of the research were exclusively provided to an entirely different group. It is worth noting that this was not a secret project run outside the bounds of peer review; rather, updates about the study’s findings were published in the *Journal of the American Medical Association* in 1936 and every half-decade or so until 1973 (Tobin, 2022); as such, this atrocity cannot be blamed solely on a rogue group of scientists. The variables responsible for the unethical treatment of the research participants in this case needed to be addressed in a systematic manner that considered the full context in which the research occurred.

#### Tudor Stuttering Study (The “Monster Study”) (1939)

In the early 1900s, Wendell Johnson, a faculty member at the University of Iowa in the USA, ran an established research lab that evaluated variables related to stuttering (Ambrose & Yairi, 2002). In 1939, a graduate student of his, Mary Tudor, conducted a study at a nearby orphanage to answer questions about whether stuttering could be induced by telling children that they had speech disfluencies. The results suggested that, at least in some cases, individuals with typical speech patterns could develop stuttering behaviors after being told that they demonstrated pathological speech tendencies. Similar to the Little Albert study, this study raises questions about the ethics of establishing problematic behaviors where previously none existed and not planning to reverse any potential harmful effects. This is particularly peculiar in this situation because Johnson, the thesis advisor, had a known history of stuttering himself and was well-acquainted with the social and communication issues associated with stuttering (Reynolds, 2006).

The advisor–graduate student dynamic presents another issue. Did Tudor have the proper competency and oversight to carry out such a study (Ambrose & Yairi, 2002)? Or, if Tudor had apprehensions about the procedures, would she have had the ability to halt or alter the study within the power differential of the advisor–advisee relationship? There is written evidence to suggest that Tudor voiced some concerns to Johnson during and after the study and that Johnson seems to have buried the results after the study was completed (Ambrose & Yairi, 2002). This prompts important questions about responsibility, competence, training, and supervision in graduate student research activities.

### Criminal Research Activities Related to World War II (mid-1930s–1947)

#### Nazi Experimentation (mid-1930s–1945)

One of the many atrocities of World War II was the medical experimentation conducted on prisoners by physicians at Nazi camps. According to the U.S. Holocaust Memorial Museum (2006), the types of unethical medical experimentation undertaken by Nazis included life-threatening research about topics such as altitude limits of parachuting, treatments for hypothermia, ways to produce potable water, methods for treating lethal injuries and illnesses through inflicting such ailments on otherwise healthy individuals, and techniques for mass sterilization and eugenics. Furthermore, when individuals died in the course of this research or for other reasons at the camps, their bodies often then were violated for experimentation as well (Weindling et al., 2016). Although the full death toll and count of victims of Nazi research may never be known for certain, some researchers have reported that, at a minimum, there were over 15,000 confirmed victims but the actual number likely is more than double that figure (Weindling et al., 2016, p. 1). It is clear that these horrific acts carried out in the name of research highlight several violations of human rights. The prisoners were subjected to deadly conditions with absolutely no benefit to them, and they were included in the experimentation involuntarily.

#### Nuremberg Medical Trial (the Doctors’ Trial) and the Nuremberg Code (1946–1947)

In late 1946, criminal proceedings were brought against 23 individuals for their alleged roles in the aforementioned Nazi experimentation; these proceedings came to be known as the “Nuremberg Medical Trial” or the “Doctors’ Trial” (U.S. Holocaust Memorial Museum, n.d. a). As the court considered the occurrence of criminal acts, research ethics suddenly held a spotlight on a worldwide stage. Given the lack of standardized research norms or written policies, Leo Alexander, a physician who was working with the prosecution, drafted a memo in April 1947 that included six requirements for “legitimate research” (U.S. Holocaust Memorial Museum, n.d. b). These requirements were reframed and augmented in the August 1947 verdict of the trial (Nuremberg Military Tribunals, 1949, pp.181–184). The updated list of 10 points became known as the “Nuremberg Code,” which described the requirements for “Permissible Medical Experiments” (hereafter referred to as the Nuremberg Code).

It should be noted that the Nuremberg Code was written to address research in general—not solely as a retrospective condemnation of the Nazi experimentation (Weindling, 2022). As such, the Nuremberg Code became a foundational ethics document upon which future documents and policies were designed. The Nuremberg Code can be found in its entirety on the U.S. Holocaust Memorial Museum’s website (U.S. Holocaust Memorial Museum, n.d. b). Due to the far-reaching formative importance of the document, the ten basic principles are included in Table 1 for easy review by readers.

**Table 1:** The Ten Principles Listed in the Nuremberg Code (adapted from United States Holocaust Memorial Museum, n.d.-b)

#	Principle
1	The voluntary consent of the human subject is absolutely essential.
2	The experiment should be such as to yield fruitful results for the good of society, unprocurable by other methods or means of study, and not random and unnecessary in nature.
3	The experiment should be so designed and based on the results of animal experimentation and a knowledge of the natural history of the disease or other problem under study that the anticipated results will justify the performance of the experiment.
4	The experiment should be so conducted as to avoid all unnecessary physical and mental suffering and injury.
5	No experiment should be conducted where there is an a priori reason to believe that death or disabling injury will occur; except, perhaps, in those experiments where the experimental physicians also serve as subjects.
6	The degree of risk to be taken should never exceed that determined by the humanitarian importance of the problem to be solved by the experiment.
7	Proper preparations should be made and adequate facilities provided to protect the experimental subject against even remote possibilities of injury, disability, or death.
8	The experiment should be conducted only by scientifically qualified persons. The highest degree of skill and care should be required through all stages of the experiment of those who conduct or engage in the experiment.
9	During the course of the experiment the human subject should be at liberty to bring the experiment to an end if he has reached the physical or mental state where continuation of the experiment seems to him to be impossible.
10	During the course of the experiment the scientist in charge must be prepared to terminate the experiment at any stage, if he has probable cause to believe, in the exercise of the good faith, superior skill and careful judgment required of him that a continuation of the experiment is likely to result in injury, disability, or death to the experimental subject.

### Mid-Century Behavioral and Medical Research with Human Participants (1950s–1998)

With the development of the Nuremberg Code, expectations for research ethics with human participants were established within the verdict of the trial, but it would take a few more decades until the principles were codified and applied to medical and behavioral research activities broadly. Even though the Nuremberg Code was available to researchers in the mid-20^th^ century, harmful research practices continued. Once again, this section is not meant to provide an exhaustive list nor are the summaries meant to fully describe the circumstances or impact of each situation. Rather, these synopses provide a sample of some research that has occurred more recently and highlight the risks of research occurring without standardized expectations and oversight.

#### Willowbrook State School Hepatitis Studies (1950s–1970s)

Willowbrook State School was a residential facility in Staten Island, New York, USA, that was established to serve children with disabilities (Goode et al., 2013). Willowbrook is well-known for extreme abuses and deplorable conditions that were made public by Senator Robert Kennedy, journalist Geraldo Rivera, and others in the 1960s and early 1970s. However, germane to this article, Willowbrook also was the site of an expansive research program. Chapter 2 of Goode et al. (2013) provides an overview of the types of medical and behavioral research that were undertaken on the children at Willowbrook, including research about hepatitis and other infectious diseases in response to outbreaks at the facility. Under the direction of Saul Krugman, hundreds of children who did not have hepatitis were infected with the virus when Krugman fed them hepatitis samples collected from the feces of infected children (Goode et al., 2013). Once again, research activities were being conducted with a vulnerable population and exposing them to a high level of risk (almost certain infection with hepatitis) with little to no likelihood of direct benefit to the participants themselves.

Krugman defended his work by stating, among other rationales, that he had obtained consent from the parents of the children. Goode et al. (2013) noted that there is reason to assume that parents were led to believe that consenting to participation in research activities would move their children to the top of Willowbrook’s long waiting list and may not have thoroughly understood the risks involved. Thus, even if parents signed Kruger’s forms, it is likely that the consent process involved coercion with the contingent offer of admission. This also highlights the tenuous boundaries of clinical activities and research activities and potential conflicts of interest when working in a clinical research context.

#### Henrietta Lacks and Her HeLa Cells (1951–ongoing)

The story of Henrietta Lacks, a Black woman who sought care for cervical cancer at Johns Hopkins University in the USA in the 1950s, is one that still is unresolved and at the center of current medical research ethics controversies (Henrietta Lacks: Science Must Right a Historical Wrong [Editorial], 2020). During the course of the medical care Lacks received, cancerous cells were extracted and studied in a laboratory. Researchers noticed that her cells had the unique ability to “survive and reproduce; they were, in essence, immortal,” (Henrietta Lacks: Science Must Right a Historical Wrong [Editorial], 2020, p. 7). Unbeknownst to Lacks or her family, the cells, named HeLa cells in the research community, were shared widely and have been used in the development of thousands of medical discoveries around the world.

The history of HeLa cells brings up several research ethics questions and concerns. First, the cells were shared without the knowledge or consent of the individual. Second, compensation, acknowledgement, or limits on the bounds of use were never discussed. Third, privacy was not adequately considered or protected. Her cells, which contain extremely personal genetic information, are in the public domain—violating not only her privacy but also the medical privacy of her descendants. As recently as 2013, the full details of a genome sequence of a HeLa strain were posted online and sparked a controversy about the privacy of the living descendants of Lacks (Greely & Cho, 2013). Although HeLa cells continue to be used in medical research, including in the development of Coronavirus Disease (COVID-19) vaccines (Henrietta Lacks: Science Must Right a Historical Wrong [Editorial], 2020), the ethical questions continue to linger.

#### Milgram Study of Obedience (early 1960s)

A third example of mid-century research, Stanley Milgram’s study of obedience, probably is familiar to anyone who has taken an introductory psychology course due to its infamous standing as an example of unethical research practices (Tolich, 2014). In the early 1960s, Milgram set out to study the effects of authority on obedience. He set up a situation in which research participants assumed the role of a teacher and a confederate (who the participant thought was another research participant) took the role of a learner (Milgram, 1963, 1974). The experimenter briefed the participant about how they would “teach” the other participant to correctly answer questions by delivering electric shocks after incorrect answers. Unbeknownst to the participant, no actual shocks would be delivered to the confederate learner. During the course of the session, the participant was instructed by the experimenter to administer what appeared to them to be more and more dangerous degrees of electric shocks. Even when the learner expressed extreme discomfort and the participant indicated that they wanted to stop administering shocks, the experimenter instructed the participant to continue. Over half of the participants continued to administer shocks to what, from their perspective, appeared to be a dangerous or even deadly level of shock to the learner.

Milgram (1963) discussed at length the various indices of distress demonstrated by the participants including verbal protests, sweating, nervous laughing, seizures, etc. The participants of the study were never offered follow-up support to process the distress that they experienced during the study (Tolich, 2014). The Milgram study is a reminder that the wellbeing of research participants does not only apply to physical safety. Even though the participants in the Milgram study were not being exposed to infectious diseases or taught maladaptive patterns of behavior like some of the other examples explored here, they still likely were experiencing adverse events that were the responsibility of the researchers to mitigate.

#### Rekers and Lovaas: “Behavioral Treatment of Deviant Sex-Role Behaviors in a Male Child” (1974)

Although the previous three examples have come from the medical field or psychology in general, the following situation has played out in the field of ABA and its flagship journal, *JABA*. In 1974, Rekers and Lovaas published an article in *JABA* that described a study in which the behavior of a 4-year-old boy, “Kraig,” was modified by reinforcing “masculine behaviors” and extinguishing and punishing “feminine behaviors.” This work was initiated as part of dissertation research by George Rekers while he was a doctoral student at the University of California, Los Angeles, USA, working under the supervision of O. Ivar Lovaas (Rekers, 1972). Almost immediately, contemporaneous critiques of the article and the ethics of the study were submitted to and published in *JABA* (Nordyke et al., 1977; Winkler, 1977). Nordyke et al. (1977) questioned the ethics of the selection of the target behaviors labeled as deviant and nondeviant in Rekers and Lovaas (1974) and then proceeded to systematically provide counterarguments to the four rationales described by Rekers and Lovaas. Winkler (1977, p. 549) also raised a concern about the selection of the target behaviors, writing, “The study raises a fundamental question, to whom does the therapist owe first allegiance: to the client (or in this case the client’s parents), to the therapist’s own values, or to prevailing relevant social norms?”

The study and controversy surrounding it resurfaced approximately 3 decades later after Kraig, whose real name was Kirk Andrew Murphy, died by suicide in 2003 (Johnson, 2022). Kirk’s family began speaking out in public, including with journalist Anderson Cooper (Cooper, 2011), and providing firsthand accounts of the devastating impact—both in the short and long term—of Rekers and Lovaas (1974). There was a subsequent resurgence among behavior analysts and the readership of *JABA* to condemn the actions taken by Rekers and Lovaas in the name of ABA research (Johnson, 2022). One result of this call for action was the publication of an Editor’s Note by the Society for the Experimental Analysis of Behavior (SEAB) and Linda LeBlanc, editor-in-chief of *JABA*, that made an official “Expression of Concern” about Rekers and Lovaas (1974; SEAB & LeBlanc, 2020). This expression of concern detailed harms that have resulted from Rekers and Lovaas (1974) but stopped short of retracting the article because “the available evidence does not make it clear that the original study was unethical by the standards of that day” (SEAB & LeBlanc, 2020, p. 1832). Subsequent discourse continues in the literature. Johnson (2022) makes the case that evidence does exist to support the retraction of the article. Both Capriotti and Donaldson (2022) and Conine et al. (2022) offer suggestions for what behavior analysts and the field of ABA can do now to address the harm that has been caused by Rekers and Lovaas (1974). As reparations are made, Rekers and Lovaas (1974) remains a grievous reminder that research activities can have long-term negative effects, especially in cases in which target behaviors are not selected in the best interest of the participant, when participants are not given the opportunity to accept or decline the opportunity to participate in research, and when harmful or abusive procedures are used as part of the method.

#### Wakefield’s Publication Erroneously Linking Vaccines and Autism Spectrum Disorder (1998)

Behavior analysts who work with individuals diagnosed with autism spectrum disorder (ASD) and their families likely, at some point in their career, will be asked about a link between ASD and vaccines. This myth and related misinformation that has been perpetuated for nearly two and a half decades traces back to unethical research conduct by Andrew Wakefield in the late 1990s (Davidson, 2017). Wakefield, a physician in London, United Kingdom (UK), published data suggesting that there was a causal link between the administration of the measles, mumps, and rubella (MMR) vaccine and the onset of behavioral patterns related to ASD (Wakefield et al., 1998). This link made sense to many given that signs of ASD often occur around two years of age—the same timeframe that the MMR vaccine typically is delivered. Despite a distinct lack of corroborating data or scientific replication, the theory gathered momentum among parents’ groups, mainstream media, and politicians alike (Davidson, 2017) and has been linked to vaccine hesitancy and increases in preventable outbreaks of related diseases (DeStefano & Shimabukuro, 2019).

In 2004, the editors of *The Lancet*, the journal in which Wakefield et al. (1998) had been published, released a statement announcing that “serious allegations of research misconduct” had been made in regards to Wakefield et al. (Horton, 2004, p. 820). This statement described six specific allegations including the accusation that proper ethics approval had not been granted for the study, that the participants in the study were not selected in the unbiased manner described in the article, and that Wakefield was linked in multiple ways to a legal case being made on behalf of parents who claimed their children were injured by vaccines (i.e., children of these families were included as participants, data were shared with lawyers prior to publication in *The Lancet*, and Wakefield personally received a substantial payment related to the legal case). The statement concluded that some allegations did not have supporting evidence and that other allegations could be rectified with a “course of full disclosure” published in the same issue (Horton, 2004, p. 821). However, after further investigation by other bodies (e.g., the UK General Medical Council’s Fitness to Practise Panel), the editors of *The Lancet* formally retracted Wakefield et al. (1998) in 2010 (Editors of The Lancet, 2010).

Even though the unethical conduct related to Wakefield et al. (1998) has been handled in the scientific community (e.g., the article has been retracted, subsequent studies have provided evidence that there is no link between ASD and vaccines [for sample evidence, see Taylor et al., 2014]), the consequences remain in the rest of society as vaccine hesitancy continues and the public’s distrust in science persists (DeStefano & Shimabukuro, 2019).

### Development of Foundational Research Ethics Documents and Policies (1953–1991)

The release of the Nuremberg Code arguably marked the beginning of the formalization of research ethics expectations as described in previous sections. However, the Nuremberg Code was limited in authority; it was not an official policy statement of any professional organization. In the 1950s and 1960s, steps were taken to turn the Nuremberg Code into something enforceable within professions and legal statutes.

#### The Ethics Code of the American Psychological Association (1953)

By the middle of the 20th century, the American Psychological Association (APA) was experiencing a shift in membership from primarily scientists and academics to a balance between scientists and practitioners and a shift in focus from scientific affairs to those of clinical practice (West, 2008). With this shift came the development of *Ethical Standards of Psychologists*, an ethics code for members of the APA (APA, 1953). (For an in-depth account of debate surrounding the establishment of an ethics code for the APA, see Joyce and Rankin (2010)). Section 4 of *Ethical Standards of Psychologists* included three items related to research ethics: (1) Maintaining standards of research; (2) Protecting welfare of research subjects; and (3) Reporting research results (APA, 1953). With the release of this document, members of the APA who were engaged in unethical research activities could be held accountable by their professional organization (Joyce & Rankin, 2010). Since 1953, the ethics code of the APA has undergone numerous revisions into the *Ethical Principles of Psychologists and Code of Conduct* that exists today (hereafter referred to as the APA Code; APA, 2017). The current APA Code, which can be found on the APA website, includes an introduction, a preamble, five general principles, and specific ethical standards, including 15 items in “Section 8: Research and Publication” regarding ethical practice in research activities.

#### Declaration of Helsinki (1964)

Following the human rights violations carried out in the name of medicine during World War II, the World Medical Association (WMA) was founded in 1947 as a global association linking national medical associations in order to set international standards in the medical profession (Ashcroft, 2008). At the annual General Assembly of the WMA in Helsinki, Finland in 1964, the membership adopted the *Declaration of Helsinki—Ethical Principles for Medical Research Involving Human Subjects* (hereafter referred to as the Declaration of Helsinki), which outlined guidance for researchers undertaking medical research activities (WMA, 2013). The Declaration of Helsinki drew heavily from the content of the Nuremberg Code, the *Declaration of Geneva* (a WMA policy, based on the Hippocratic Oath, that includes a physician’s conduct pledge as a member of the medical profession; WMA, 2017), and the *International Code of Medical Ethics* (an ethics code governing the practice of medicine by members of the WMA; WMA, 2022). Central to the Declaration of Helsinki was the requirement that informed consent must be obtained prior to the initiation of research activities and that medical researchers should prioritize a patient or participant’s welfare over any research ambitions. At the same time, the role of clinical research was legitimized, and physicians were encouraged to use experimental methods if there was a potential benefit for patients. The original version of the Declaration of Helsinki can be found on the WMA’s website (WMA, n.d.).

Since the first adoption in 1964, the Declaration of Helsinki has been amended or clarified nine times, most recently in 2013. This version is available in an open-access format online through the *Journal of the American Medical Association* (WMA, 2013). The current version of the Declaration of Helsinki includes 13 general principles in the first section of the document. These principles require physicians to prioritize patient wellbeing while also advancing the field of medicine with ongoing clinical research.

It is the duty of physicians who are involved in medical research to protect the life, health, dignity, integrity, right to self-determination, privacy, and confidentiality of personal information of research subjects. The responsibility for the protection of research subjects must always rest with the physician or other health care professionals and never with the research subjects, even though they have given consent. (WMA, 2013, p. 2191)

This section also requires that those conducting research with human participants must have the proper training and qualifications and that underrepresented groups must have access to participate in medical research. The next section of the Declaration of Helsinki addresses risks, burdens, and benefits and requires that the benefits must always outweigh the risks and burdens. In the subsequent section, vulnerable populations are considered and necessary protections are discussed. Other sections of the Declaration of Helsinki cover scientific requirements and research protocols; research ethics committees; privacy and confidentiality; informed consent; use of placebo; posttrial provisions; research registration, publication, and dissemination of results; and unproven interventions in clinical practice.

#### National Research Act (1974) and the Belmont Report (1979)

Although the Declaration of Helsinki was written for a medical research framework, the next decade resulted in similar advances for behavioral research. Of note, although the Declaration of Helsinki applied to an international community, the following foundational documents were developed within the context of the USA. However, the documents have been used—both formally and informally—to develop processes and policies around the globe, so understanding their context likely still is valuable for an international audience. Of course, readers conducting research outside the USA are encouraged to seek further guiding documents specific to their region.

In the early 1970s, as a result of both public and professional discussion of research ethics practices, Senator Edward Kennedy led congressional hearings about research involving human participants (Rice, 2008). These hearings concluded that the federal government had a responsibility to protect human research participants, and the National Research Act was signed into law in 1974. This law established the National Commission for the Protection of Human Subjects of Biomedical and Behavioral Research (hereafter referred to as the National Commission) to draft formative documents related to research requirements. The National Commission included “experts in ethics, religion, law, industry, and medicine” (Rice, 2008, p. 1328). Of interest to behavior analytic researchers, Joseph Brady, a pioneer in the field of behavior analysis, was the associate chairperson of the 11-person National Commission (Thompson, 2012). Thus, as research ethics standards were codified into law in the USA, a behavior analytic voice was at the table.

The result of the work of the National Commission was the issuance of *The Belmont Report: Ethical Principles and Guidelines for the Protection of Human Subjects of Research* (hereafter referred to as the Belmont Report; National Commission, 1979). The Belmont Report can be found in its entirety on the website of the Office for Human Research Protections.

The Belmont Report begins with a section about the boundaries between practice and research (Part A). Then, in Part B, the Belmont Report lays out three basic principles for ethical research with human participants: respect for persons, beneficence, and justice. Ethical requirements related to respect for persons include obtaining voluntary and informed consent, protecting confidentiality and privacy, and allowing participants to withdraw from research activities without penalty. The principle of beneficence requires that researchers minimize harm while maximizing benefit to the research participant and/or the community. The final principle, justice, requires that the burdens of research do not fall unduly to certain populations, especially vulnerable populations. Likewise, specific populations should not be excluded from research opportunities. These principles still are the pillars against which modern research is evaluated and approved or disapproved (Protection of Human Subjects, 1991). Part C of the Belmont Report covers applications of the principles to research activities and describes how researchers should obtain informed consent, assess risks and benefits, and select participants.

#### Protection of Human Subjects, 45 C.F.R. Part 46 (including the “Common Rule”) (1991)

Once the National Commission completed its tasks and the Belmont Report was finalized, the responsibility for oversight of research with human participants in the USA shifted to the Office for Human Research Protections within the Department of Health and Human Services (Rice, 2008). In 1991, the principles and applications of the Belmont Report were codified in the U.S. Code of Federal Regulations at 45 C.F.R. Part 46—Protection of Human Subjects (hereafter referred to as Part 46), which governs all research involving human participants conducted, supported, or otherwise subject to regulation by any federal department or agency (Protection of Human Subjects, 45 C.F.R. Part 46, 1991), unless specifically exempted by the Code of Federal Regulations (see Part 46, §46.101(a)). Though some revisions have been made in the past 30 years, this foundational document still regulates human participant research conducted in the USA and can be found in full on the official Code of Federal Regulations website (Protection of Human Subjects, 45 C.F.R. Part 46, 1991). Again, even though the policies were designed for and are used in the context of the USA, international readers may be interested in learning about the document because it has been used to guide research ethics regulations in other countries.

Part 46 is organized into five subparts. Subpart A, also referred to as the Common Rule, sets forth comprehensive requirements for researchers who undertake research with any human participants, including defining research activities and defining human participants. In addition, the Common Rule thoroughly describes the requirements for institutional review boards (IRBs), including the membership and procedures. (Note: The shorthand title of the “Common Rule” technically refers only to Subpart A, even though it has sometimes been erroneously used to describe the entirety of Part 46.) Subpart B describes additional protections for pregnant women, human fetuses, and neonates. Subpart C covers additional protections for prisoners. Subpart D discusses additional protections for children. This subpart also addresses requirements for the obtainment or waiver of assent. Subpart E includes requirements for registering IRBs. Overall, Part 46 serves as the guidance that IRBs in the USA should use when determining whether research with human participants should be approved for federally-funded or otherwise federally-overseen research. Thus, any researcher who is conducting such research in the USA is responsible for being familiar with and adhering to Part 46.

Of course, this primer could have included a variety of other examples of unethical research practices or could have summarized a number of other policy statements and documents, and curious readers are encouraged to continue on this historical journey by using this article’s reference list as a starting point. The purpose here was not to detail every single step along the way to the policy documents behavior analytic researchers have today but rather to contextualize contemporary policies in significant historical events and foundational documents. By understanding at least a bit of the historical context, behavior analytic researchers can identify the *why* behind the *what* of today’s research ethics standards and make links between the BACB Code (or other relevant codes or practices if they are not certified by the BACB) and the historical context.

## Linking the BACB Code to Foundational Research Ethics Documents and Significant Historical Events

As mentioned previously, the BACB Code lays out expectations for BCaBA and BCBA applicants and certificants who engage in research activities. It is worth noting that the four Core Principles of the BACB Code (benefit others; treat others with compassion, dignity, and respect; behave with integrity; ensure their competence; BACB, 2020, p. 4) are similar in content and tone to the basic ethical principles of the Belmont Report (respect for persons; beneficence; justice; National Commission, 1979) and the APA Code (beneficence and nonmaleficence; fidelity and responsibility; integrity; justice; respect for people’s rights and dignity; APA, 2017). Thus, the impact of foundational research ethics documents is not limited only to research practices; these documents had a hand in shaping much broader aspects of ethical professional activities.

As is common practice in a professional code, the requirements in the BACB Code are not specifically linked to any foundational research ethics documents or significant historical events. In this section, in order to align with the objectives of this special issue of *Behavior Analysis in Practice,* this article will draw some of those links to allow readers to consider some potential rationales for the inclusion of various BACB Code items. Each of the six items in “Section 6—Responsibility in Research” that directly address the protection of human participants will be discussed in turn. Table 2 summarizes each foundational document discussed in the previous section and links the documents to the BACB Code. The full text of the BACB Code can be found on the BACB’s website (BACB, 2020). For behavior analytic researchers who are not certified by the BACB and, therefore, whose behavior is not bound by the BACB Code, this section may be helpful in other ways (e.g., prompting links to other professional codes that govern their practice, promoting reflection on their own research activities and those of their colleagues, aiding in the development of professional codes where none exist).

**Table 2:** An Overview of Five Foundational Ethics Documents including Links to Their First and Most Recent Versions, the Documents’ Authors and Target Audience, the Documents’ Purpose and Selected Notable Features, and the Documents’ Alignment with the Core Principles and Section 6 of the Behavior Analyst Certification Board’s (BACB) Ethics Code for Behavior Analysts (2020)

Foundational Ethics Document	First Version/ Most Recent Update	Author / Origin	Audience	Purpose & Notable Features	Document Item ➔ BACB Code
Nuremberg Code	1947	Included in the verdict of the Nuremberg Medical Trial	Medical professionals	Purpose: To outline permissible activities in medical research. Notable feature: Formally introduced the concept of voluntary consent.	1 ➔ 6.04 4 ➔ CP^a^1 8 ➔ 6.06 9 ➔ 6.04, CP2
Ethical Principles of Psychologists and Code of Conduct	1953 / 2017	American Psychological Association (APA)	Psychologists (scientists and practitioners)	Purpose: To provide a common set of principles for psychologists and standards of professional conduct. Notable feature: Created a mechanism to hold psychologists professionally responsible for unethical research conduct.	Principle A ➔ CP1 Principle B ➔CP1 Principle C ➔ CP3 Principle D ➔ CP2, CP4 Principle E ➔ CP2 1.02 ➔6.01 2.01, 2.03, 2.05 ➔ 6.06 3.01 ➔ CP2 3.04 ➔ CP1 3.10, 8.02, 8.03 ➔ 6.04 4.01, 4.02, 4.06, 4.07 ➔ 6.05 5.01 ➔ 6.11 6.01, 6.02 ➔ 6.10 8.01 ➔ 6.02 8.06 ➔ 6.03 8.10 ➔ 6.11 8.11, 8.13 ➔ 6.09 8.12 ➔ 6.08
Declaration of Helsinki—Ethical Principles for Medical Research Involving Human Subjects	1964 / 2013	World Medical Association (WMA)	Physicians Others who are involved in medical research involving human participants	Purpose: To guide individuals undertaking medical research activities. Notable feature: Section dedicated to vulnerable groups and individuals (19–20) Notable feature: Outlines necessary components in a research protocol (22)	4 ➔ CP1, 6.03 7 ➔ CP1, CP2 9 ➔ CP2 10 ➔ 6.01 12 ➔ 6.06 14 ➔ 6.03 23 ➔ 6.01, 6.02 24 ➔ 6.05 25-32 ➔6.04 36 ➔ 6.07, 6.11
The Belmont Report: Ethical Principles and Guidelines for the Protection of Human Subjects of Research	1979	The National Commission for the Protection of Human Subjects of Biomedical and Behavioral Research	Researchers, members of Institutional Review Boards, and federal employees	Purpose: To identify the basic ethical principles that should underlie the conduct of biomedical and behavioral research involving human participants and to develop guidelines which should be followed to assure that such research is conducted in accordance with those principles. Notable feature: Outlines requirement for fair procedures and outcomes in the selection of research participants.	Part A ➔ 6.03 Part B1, B3 ➔ CP2 Part B2 ➔ CP1 Part C1 ➔ 6.04
Protection of Human Subjects, 45 C.F.R. Part 46	1991	Part of the U.S. Code of Federal Regulations	Individuals and institutions involved in federally-funded / federally-overseen research in the United States	Purpose: Policy applies to all research involving human participants conducted, supported, or otherwise subject to regulation by any U.S. federal department or agency unless specifically exempted by the Code of Federal Regulations. Notable feature: Subpart A thoroughly describes the requirements for IRBs, including the membership and procedures. Notable feature: Subpart D outlines guidelines for soliciting the assent of the children.	§ 46.107 -§ 46.115 ➔ 6.02 § 46.116-§ 46.119 ➔6.04

^a^CP denotes Core Principle

### 6.01 – Conforming with Laws and Regulations in Research

Behavior analysts plan and conduct research in a manner consistent with all applicable laws and regulations, as well as requirements by organizations and institutions governing research activity. (BACB, 2020, p. 17)

In order to follow BACB Code item 6.01, behavior analysts must be familiar with laws and regulations governing research. As described above in cases, such as the Willowbrook State School experiments, the outcomes of unregulated research can be disastrous and deadly. After learning what was happening in the name of research prior to comprehensive laws and regulations, behavior analytic researchers should understand why the policies were developed and why the protections are a critical component of the research process.

The primer of foundational documents included in this article may be a good place to begin, but researchers are encouraged to return to the documents themselves and not rely on third-party summaries. At a minimum, behavior analytic researchers in the USA should be familiar with the Belmont Report and Part 46. Researchers working in other countries may want to return to the Nuremberg Code and the Declaration of Helsinki in addition to determining what laws and regulations apply to their setting.

### 6.02 – Research Review

Behavior analysts conduct research, whether independent of or in the context of service delivery, only after approval by a formal research review committee. (BACB, 2020, p. 17)

The significant historical events described previously (e.g., Little Albert research, Tudor stuttering study, Milgram study of obedience, Wakefield vaccine publication) demonstrated the critical need for formal research review, approval, and oversight. The same person developing and running a study should not be the same person determining whether the study meets the necessary ethical requirements.

Several foundational ethics documents provide substantial guidance regarding requirements for a research review committee. In particular, the Belmont Report and Part 46 describe exactly how to set up an IRB and how the IRB should function. Researchers who are not conducting research that is funded or otherwise overseen by the USA’s federal government may also want to review sources that offer guidance about conducting research in clinical contexts and setting up ethics committees in non-university settings (e.g., Cox, 2020; LeBlanc et al., 2018; Normand & Donohue, 2023).

### 6.03 – Research in Service Delivery

Behavior analysts conducting research in the context of service delivery must arrange research activities such that client services and client welfare are prioritized. In these situations, behavior analysts must comply with all ethics requirements for both service delivery and research within the Code. When professional services are offered as an incentive for research participation, behavior analysts clarify the nature of the services, and any potential risks, obligations, and limitations for all parties. (BACB, 2020, p. 17–18)

Significant events in the history of medical research provide modern researchers with many tales of caution regarding conducting research in a clinical setting. Stories such as those about Henrietta Lacks and the Willowbrook State School remind researchers of the importance of prioritizing client wellbeing over any possible research outcomes. Readers who are interested in diving deeper into the ethics of clinical research are advised to read the full Declaration of Helsinki to understand how the medical field addresses research conducted in the course of service delivery. Part A of the Belmont Report also covers the boundaries between clinical practice and clinical research while Subpart D of the Part 46 discusses the ethics of enrolling children into clinical research studies when the child otherwise would not be able to access the treatment or intervention. Finally, Normand and Donohue (2023) provide a thorough discussion of research in clinical service delivery in the field of behavior analysis as it relates to the BACB Code.

### 6.04 – Informed Consent in Research

Behavior analysts are responsible for obtaining informed consent (and assent when relevant) from potential research participants under the conditions required by the research review committee. When behavior analysts become aware that data obtained from past or current clients, stakeholders, supervisees, and/or trainees during typical service delivery might be disseminated to the scientific community, they obtain informed consent for the use of the data before dissemination, specify that services will not be impacted by providing or withholding consent, and make available the right to withdraw consent at any time without penalty. (BACB, 2020, p. 18)

The idea of informed consent goes all the way back to the atrocities and human rights violations committed during World War II under the name of research and the ensuing description of voluntary consent in the Nuremberg Code. A common thread among many of the significant historical events summarized here is that participants were not informed of the procedures and potential benefits and risks of the research activities and/or were not given the opportunity to decide whether or not they wanted to be part of the studies (e.g., Tuskegee syphilis study; Rekers & Lovaas (1974)).

Readers are encouraged to read the Nuremberg Code in full to understand the original spirit of informed consent. Then, Subpart A of Part 46 should be consulted to determine the current requirements for a participant’s agreement to be considered informed consent. Subpart D of Part 46 provides guidance to researchers and reviewers regarding when and how assent should be obtained during research.

### 6.05 – Confidentiality in Research

Behavior analysts prioritize the confidentiality of their research participants except under conditions where it may not be possible. They make appropriate efforts to prevent accidental or inadvertent sharing of confidential or identifying information while conducting research and in any dissemination activity related to the research (e.g., disguising or removing confidential or identifying information). (BACB, 2020, p. 18)

The major violation to Henrietta Lacks is a prime example of what can happen if researchers do not prioritize participants’ confidentiality. Due to lack of privacy protections, an exceptional amount of her private details, including her genetic information and that of her relatives, were released into the public domain. As contemporary researchers are preparing their procedures, the Common Rule (Subpart A of Part 46) should be consulted. The Common Rule advises IRBs that they should require adequate protections for participants’ confidentiality before approving a study and requires that those protections be included in the informed consent process. In addition, the Common Rule addresses what private information is and how identifiable private information should be protected.

### 6.06 - Competence in Conducting Research

Behavior analysts only conduct research independently after they have successfully conducted research under a supervisor in a defined relationship (e.g., thesis, dissertation, mentored research project). Behavior analysts and their assistants are permitted to perform only those research activities for which they are appropriately trained and prepared. Before engaging in research activities for which a behavior analyst has not received training, they seek the appropriate training and become demonstrably competent or they collaborate with other professionals who have the required competence. Behavior analysts are responsible for the ethical conduct of all personnel assigned to the research project. (BACB, 2020, p. 18)

The case of the Tudor stuttering study potentially is an example of what might happen if adequate supervision is not provided to novice researchers. Although no one will ever know if the study would have progressed as far as it did if Mary Tudor’s advisor had had tighter oversight of the research activities, the decision of whether to continue or terminate the study would likely not have been left on the shoulders of a 22-year-old graduate student if regulations, such as BACB Code item 6.06, were in place at the time. By the issuance of the Nuremberg Code, it was clear that researcher competence was a necessary component of ethical research, and the Declaration of Helsinki and APA Code further described requirements for training and qualifications to oversee research with human participants. Behavior analysts also may want to consult Brodhead et al. (2018) for a discussion of scope of competence in behavior analysis. 

## Sample Scenarios in which Foundational Research Ethics Documents May Provide Guidance

Although one potential benefit of being familiar with significant historical events and foundational documents in research ethics is understanding the *why* behind contemporary practices, a second—yet related—potential benefit may have more practical utility to behavior analytic researchers. In situations in which the BACB Code does not provide explicit instruction or when the behavior analytic researcher is not bound by the BACB Code (i.e., the researcher is not certified by the BACB), a familiarity with foundational documents may be helpful insofar as those documents can provide guidance. Of note, this should not be interpreted as a limitation or flaw of the BACB Code. Rather, the BACB Code was written in a manner that guides certificants and applicants to return to the framework (i.e., the Core Principles; BACB, 2020) when making decisions about any aspect of their work. Of course, a single code cannot detail every possible situation that may arise during the course of activities, so it is incumbent on those bound by the BACB Code to use the Core Principles and foundational documents to interpret and generalize the standards in the BACB Code to any novel circumstances.

In this section, five hypothetical scenarios are described and examples of how foundational documents could be consulted are provided. This is not meant to be an exhaustive list of circumstances; rather, these scenarios were designed to serve as a model of how to apply foundational documents to behavior analytic research contexts.

### Scenario 1: Recruitment and Selection of Participants

Rachel is a BCBA working in a public school in the USA. She is interested in conducting research regarding the most effective ways to teach handwashing to early elementary school students. She runs a free after school program for children from families at or below 130% of the federal poverty level. She is considering inviting all students in the afterschool program to participate because she already has access to these students and these students have more availability due to their extended hours at the school. However, she is wondering if this is an ethical approach to recruitment into a research project, and the BACB Code does not mention recruitment strategies explicitly.

Rachel could consult either the Declaration of Helsinki or the Belmont Report. Both documents would caution her against this recruitment approach. If Rachel proceeded with only including students from the afterschool program, she runs the risk of unduly burdening one specific group, a potentially vulnerable group, during the creation of knowledge that likely would benefit other groups. If a vulnerable group is going to be considered for inclusion in a study, the Declaration of Helsinki requires that the research be “responsive to the . . . needs or priorities of the group and the research cannot be carried out in a non-vulnerable group” (WMA, 2013, p. 2192). The Belmont Report’s discussion of justice also might help Rachel develop more equitable recruitment strategies. She would discover that ease of access to participants is not an ethical reason to target a specific group for inclusion in a study. (See Pritchett et al., 2021, for further discussion about moving from colonial to participatory research practices in the field of behavior analysis.)

### Scenario 2: Obtaining Assent

Aisha is a Board Certified Behavior Analyst-Doctoral® (BCBA-D) who runs a severe behavior outpatient clinic on a university campus. Clients are referred to her for the assessment of extremely dangerous behavior, and she is evaluating a slight modification to typical assessment procedures in order to determine if the modification would result in a more efficient protocol. The BACB Code mentions the obtainment of assent in 6.04 (“Behavior analysts are responsible for obtaining informed consent (and assent when relevant)”; BACB, 2020, p. 18). In addition, assent is defined in the BACB Code as

Vocal or nonvocal verbal behavior that can be taken to indicate willingness to participate in research or behavioral services by individuals who cannot provide informed consent (e.g., because of age or intellectual impairments). Assent may be required by a research review committee or a service organization. In such instances, those entities will provide parameters for assessing assent. (BACB, 2020, p. 7)

However, Aisha is not sure how to interpret “when relevant” in her situation.

Aisha may consider reviewing the Belmont Report and Subpart D of Part 46. Although the decision ultimately will rest with Aisha and her research team, the IRB approving the study, and the guardians responsible for providing consent for the potential participants, foundational documents could help in the drafting of the proposed procedures. In Part B, the Belmont Report discusses the importance of prioritizing autonomy and protecting individuals with diminished autonomy in the ethical principle of respect for persons. Subpart D details conditions under which assent may be waived (e.g., extremely limited capacity for consultation, the research activities offer an intervention that is not accessible any other way and is likely to benefit the individual directly). After Aisha scrutinizes her project in relation to foundational documents, she may also turn to contemporary peer-reviewed literature for ideas about how meaningful assent might be obtained if it is determined that assent is appropriate in her situation (e.g., Mead Jasperse et al., 2023; Morris et al., 2021; Rajaraman et al., 2022).

### Scenario 3: Compensation for Participation in Research

Felipe is a BCBA who is developing a research proposal to evaluate a behavioral intervention to teach school bus drivers how to complete a safety routine at the end of their route. Felipe understands that his participants are very busy and thinks that they should be appropriately compensated for their valuable time. He also thinks that including an incentive could increase the likelihood of successful recruitment. He is wondering if he can include gift vouchers in his funding request, and, if so, how to decide the value of the vouchers. Felipe is aware that the BACB Code indicates what to do regarding the exchange of gifts in a practice setting (BACB, 2020, p. 10), but he is not sure what to do when conducting research.

Felipe might take a look at the Belmont Report as he develops his budgetary requirements for this research project. The Belmont Report states in Part C.1 that “Undue influence . . . occurs through an offer of an excessive, unwarranted, inappropriate or improper reward or other overture in order to obtain compliance” (National Commission, 1979). Therefore, although the provision of incentives is allowed, Felipe needs to carefully consider the amount offered to “minimize the possibility of coercion or undue influence,” as per Section 116 of Part 46 (Protection of Human Subjects, 1991). Felipe should consider all aspects of the research requirements when proposing the compensation value and must ensure that the vouchers are not worth so much that they induce a potential participant to consent when they would not otherwise think that the benefits outweigh the risks of participation.

### Scenario 4: Selection of Target Behaviors

Alfie is a BCBA-D who works as an assistant professor and runs a research lab at their university. They are interested in studying the impact of schedules of reinforcement on reading skills and want to do this by manipulating schedules while teaching participants to read nonsense words aloud and match the words with their made-up meaning. They plan to recruit adult participants only and do not plan to use any deception (i.e., the informed consent process will detail exactly what will occur during the research sessions). However, they’re concerned about selecting a target behavior that does not have the potential to have a direct positive impact on the participants, and the BACB Code doesn’t explicitly mention the selection of target behaviors when conducting research outside the context of service delivery.

Alfie might want to return to Subpart A of Part 46 and review the requirements for informed consent (§46.116). Although Alfie’s target behavior appears fairly benign (maybe even fun if a participant enjoys memory and learning games!), there could be instances in which the target behavior might have the potential to cause harm due to individual circumstances (e.g., the participant has struggled with reading acquisition in the past and aversive emotions are associated with the task). Thus, the informed consent process will be critical in terms of providing potential participants with enough information about the study such that they can determine whether there are any potential adverse effects they could experience. With a study such as this, one burden that may exist for all participants is that of the time commitment. That is, participants are going to spend time doing an activity that does not apparently have any direct benefit to themselves. As such, guided by Subpart A, Alfie’s consent form should include a clear statement of the estimated time involved and a statement that participation is voluntary and participants can withdraw at any point without penalty. Finally, even though the informed consent process should empower participants to make their own choices regarding participation, Alfie should remember the last item in the Nuremberg Code—the researcher must take responsibility for the experiment and be ready to terminate the study at any point if the participant is at risk.

### Scenario 5: Research Involving Deception

Rashid is a BCBA and a graduate student in a doctoral program. As part of his dissertation research, he is interested in identifying ways to teach preteens to stay safe when engaging on social media, including not sharing personal information on the platform, not accepting or making friend requests to people they do not know offline, and not joining any groups that are not designed for teens and moderated appropriately. He wants to test a behavioral skills training (BST) model, but his project is going to need to involve a bit of deception to determine if BST has been effective. In particular, his plan is to have one of his research assistants pose as an age-matched peer on the social media platform and observe how the preteen participant responds to various situations including requests for personal information, friend requests, etc. Rashid knows that the BACB Code clearly requires informed consent (and assent, as appropriate) in 6.04, but he’s not sure how to handle the informed consent and assent process when deception is being proposed.

Once again, a return to Part 46 might be helpful in this situation. Rashid could take a look at §46.116f that describes circumstances in which a general waiver or alteration of consent might be warranted. If he can determine that the research involves no more than minimal risk to the participants, that the research could not be carried out without the requested alteration (in this case, the deception to the preteens regarding who they are interacting with online), that the participants’ rights and welfare will not be compromised, and that the participants will be fully debriefed after the study, then he may be able to make the case for deception within the assent process. Rashid may consider including the aspect of deception in the assent process (but not details about exactly what the deception will be) and should thoroughly disclose the nature of the deception in the informed consent process with the parents/guardians. Rashid also may want to turn to standard 8.07 in the APA Code as it delineates requirements for involving deception in research by psychologists that may be helpful for Rashid’s context.

These five scenarios provide models of how behavior analytic researchers can use foundational documents to inform their practices when the BACB Code does not offer explicit guidance. Of course, these are not the only novel dilemmas that could occur when designing behavior analytic research protocols. Table 3 organizes the dilemmas presented in this article as well as additional research ethics concerns and suggests related foundational ethics documents and peer-reviewed articles that might offer direction when such concerns are encountered.

**Table 3:** Sample Research Ethics Concerns and Suggested Related Foundational Ethics Documents and Peer-Reviewed Articles

Research Ethics Concerns	Suggested Resources
Requirements for Institutional Review Boards (IRBs)	Belmont Report (1979) Protection of Human Subjects, 45 C.F.R. Part 46 (1991: • Subpart A*—*membership and procedures • Subpart E*—*requirements for registering IRBs
Conducting research in clinical contexts and setting up ethics committees in non-university settings	Cox (2020) LeBlanc et al. (2018) Normand & Donohue (2023)
Boundaries between clinical practice and clinical research	Declaration of Helsinki (2013) Belmont Report (1979)
Enrolling children and vulnerable populations into research	Declaration of Helsinki (2013) Belmont Report (1979) Protection of Human Subjects, 45 C.F.R. Part 46 (1991): • Subpart D
Understanding, assessing, and minimizing risk	Nuremberg Code Protection of Human Subjects, 45 C.F.R. Part 46 (1991) LeBlanc et al. (2018) Deochand et al. (2020)
Recruitment and selection of participants	Declaration of Helsinki (2013) Belmont Report (1979) Pritchett et al. (2021)
Selection of target behaviors	Nuremberg Code Protection of Human Subjects, 45 C.F.R. Part 46 (1991): • Subpart A Bosch & Fuqua (2001)
Research involving deception or waiver/alteration of consent	Protection of Human Subjects, 45 C.F.R. Part 46 (1991) APA Code (Standard 8.07, 2017)
Obtainment or waiver of assent	Protection of Human Subjects, 45 C.F.R. Part 46 (1991): • Subpart D Belmont Report (1979) Morris et al. (2021) Flowers & Dawes (2023) Rajaraman et al. (2022)
Compensation for participation in research	Belmont Report (1979): Part C Protection of Human Subjects, 45 C.F.R. Part 46 (1991) APA Ethics Code (2017)

*Note: *This list of research concerns and suggested resources is not (and was not intended to be) comprehensive. We acknowledge that behavior analytic researchers may face other research ethics situations not outlined in this table

The foundational documents and historical events also might be useful at an agency level. For example, behavior analytic organizations that primarily deliver clinical services might be interested in developing policies to guide clinical research conducted at their facility. In this case, in addition to reading Cox (2020), LeBlanc et al. (2018), and Normand and Donohue (2023), the policymakers at the agency might want to consult foundational documents as they create their own guiding documents. As an example, an agency might want to design a policy about the required documents that a scientist-practitioner must submit in order to apply to conduct research at their organization. Besides mentioning that informed consent must be obtained, the BACB Code does not stipulate any required documents or applications to conduct research as a BACB certificant. In this case, the Declaration of Helsinki might be helpful. In Section 22, the Declaration of Helsinki lists the required components of a research protocol, including the research design and ethical considerations, such as funding, potential conflicts of interest, incentives for participants, and provisions for participants who are harmed in the course of the study. This is only one example of how agency leaders might utilize foundational documents and historical events to design policies, procedures, and processes related to clinical research. Additional organization-level requirements that could be guided by these resources include tasks such as establishing expectations for the selection, training, and oversight of a research ethics committee, defining equitable selection of research participants, determining how to minimize risk and maximize benefit in clinical research, identifying how to ethically bill for clinical research activities, planning for ethical dissemination of research findings, and outlining the expectations for research involving staff, parents, caregivers, or other stakeholders (e.g., parent survey research or staff training research). One helpful next step might be the publication of a resource for policymakers that makes explicit recommendations for research policy development based on guidance from foundational ethics documents.

## Future Directions for Data-Driven Decision-Making in Research Ethics

In alignment with the theme of this special issue of *Behavior Analysis in Practice*, the final section of this article offers some ideas for ways in which behavior analytic researchers might collect and use data to make decisions about research ethics in alignment with the BACB Code and foundational documents.

### What Research Ethics Data Are Available Now?

Before looking to future directions in data-driven research ethics decisions, a consideration of what research ethics data are available now might be useful. One place to start would be an analysis of research-related violations of BACB Code items, including previous versions of the professional and ethical guidelines set forth by the BACB. The BACB has a dedicated webpage for publishing reportable sanctions the BACB has taken against an individual’s certification or application eligibility dating back to 2002 (BACB, 2023a). These sanctions also appear in the BACB Certificant Registry (BACB, 2023b). As of January 22, 2023, disciplinary actions are publicly listed for the United States (*n* = 1,025), Canada (*n* = 1), and China (*n* = 1). From an analysis of this database, there have been no published disciplinary actions for research-related ethics standards.

Given that research activities often are construed as having a higher likelihood of involving risk than nonresearch activities, this is an interesting finding that would be worthy of investigation on its own. What variables are responsible for this data point? Are the pre-approvals that are required by IRB and research review committee processes effectively safeguarding against unethical research activities? Or, are research ethics violations either not being reported or not reaching the level of reportable sanctions? Or, maybe are research activities being undertaken and supervised by a subset of certificants who have relatively more training and experience? Whatever the reason(s), this big-picture data point is one that might be worthy of following as research ethics decisions are made in the field of behavior analysis.

### What Data Might Be Helpful at the Group (Field) Level?

BACB Code item 6.06 discusses competence in conducting research. As the field of behavior analysis grows, data regarding training opportunities in research ethics might be useful. This could take the form of collecting data regarding continuing education events covering research ethics. BACB certificants already are required to report how many continuing education units (CEUs) they acquire in the area of ethics (BACB, 2022), so data collection might take the form of adding a box to tick if the ethics CEU addressed research ethics. As an alternative, approved continuing education (ACE) providers could be asked to report annually how many CEU events were on the topic of research ethics. These data and any similar data collected by other certifying bodies might help the field collectively make decisions about whether more resources should be dedicated to designing and offering research ethics CEUs. In a similar vein, behavior analysts might be interested in learning how research ethics are being taught in graduate coursework (e.g., in a standalone ethics course, in a research methodology course, integrated across courses). These data could be collected by surveying Verified Course Sequence coordinators about the incorporation of research ethics into their curriculum.

As a field, behavior analysts also might want to know how many BACB certificants are engaged in research activities. Data regarding the proportion of certificants conducting research might help to interpret other group-level data (e.g., the absence of research-related ethics violations) and plan for future growth and needs of the field. These data might be collected by asking BACB certificants to self-report research activities with a binary yes/no question during the recertification process.

Finally, research regarding various research ethics activities in the field of behavior analysis might be helpful. This could take the form of reviewing published literature (e.g., Morris et al., 2021), collecting self-report data via survey research (e.g., Mead Jasperse et al., 2023) or interviews/focus groups, or empirically evaluating the utility of specific procedures related to research ethics (e.g., Rajaraman et al., 2022). As these data sets are generated, more informed decisions regarding research ethics policies and practices can be made at the group level.

### What Data Might Be Helpful at the Individual Scientist-Practitioner Level?

The foundational documents reviewed in this article offer numerous ideas for data collection to objectively determine adherence or nonadherence to a high standard of research ethics. For example, Part 46 requires that IRBs have written protocols that researchers are to follow regarding unanticipated problems involving risk to participants or others. Researchers may want to develop objective systems for tracking data related to risks, research-related harm or injuries, or unexpected problems.

Another type of data that might be useful to monitor to ensure compliance with research ethics policies would be recruitment and demographic data. In 2017, Li et al. brought this issue forward for consideration and made the case that reporting demographic information is necessary to determine if the principle of justice from the Belmont Report is being carried out. However, when Jones et al. (2020) conducted an updated review of the literature a few years later, they found that demographic variables were still being underreported. Thus, a reiterated call for collecting and reporting demographic data in order to determine adherence to research ethics guidelines might be warranted.

Finally, as appropriate, behavior analytic researchers might want to consider collecting data on and documenting ongoing assent in research contexts, such as Rajaraman et al. (2022) did. Collecting these data would allow researchers to calculate the proportion of scheduled sessions that were assented to versus those that were not assented to, which might in turn help researchers make data-driven decisions regarding whether a participant should be considered for continued participation in a study.

## Research Ethics in Behavior Analysis in 2023 and Beyond

“It isn’t a question of starting. The start has been made. It’s a question of what’s to be done from now on” (Skinner, 1948, p. 208).

This primer of significant historical events and the development of foundational research ethics documents began in the early 1920s. As summarized in this article, the past century has borne witness to both horrific atrocities that have occurred in the name of research as well as incredible progress in terms of formalization and codification of research ethics standards. With the BACB Code coming into effect on January 1, 2022 (BACB, 2020), there is great opportunity for behavior analytic researchers to lead continued progress in the area of research ethics. An understanding of the historical variables responsible for current policies and practices may allow behavior analytic researchers to better understand the potential rationale for the inclusion of various BACB Code items. Furthermore, familiarity with the foundational research ethics documents may help behavior analysts generalize the Core Principles of the BACB Code to novel research ethics situations and in cases in which behavior analytic researchers are not certified by the BACB. As behavior analytic researchers push forward into the next century of progress in research ethics, the data collected at both the group and individual level may help the field make informed decisions about the next iterations of research ethics policies and practices.

## Data Availability

Data sharing not applicable to this article as no data sets were generated or analyzed during the current study.

## References

[CR1] Ambrose, N. G., & Yairi, E. (2002). The Tudor study: Data and ethics. *American Journal of Speech-Language Pathology,**11*, 190–203. 10.1044/1058-0360(2002/018)

[CR2] American Psychological Association. (1953). *Ethical standards for psychologists*. https://www.apa.org/pubs/books/supplemental/Essential-Ethics-Psychologists/1953ethicscode.pdf. Accessed 17 Oct 2023

[CR3] American Psychological Association. (2017). *Ethical principles of psychologists and code of conduct* (2002, amended effective June 1, 2010, and January 1, 2017). https://www.apa.org/ethics/code/

[CR4] Ashcroft, R. E. (2008). The Declaration of Helsinki. In E. J. Emanuel, C. Grady, R. A. Crouch, R. K. Lie, F. G. Miller, & D. Wendler (Eds.), *The Oxford textbook of clinical research ethics* (pp. 141–148). Oxford University Press.

[CR5] Behavior Analyst Certification Board. (2020). *Ethics code for behavior analysts*. https://bacb.com/wp-content/ethics-code-for-behavior-analysts/

[CR6] Behavior Analyst Certification Board. (2022). *Board certified behavior analyst* *handbook*. https://www.bacb.com/bcba-handbook/

[CR7] Behavior Analyst Certification Board. (2023a). *BACB disciplinary actions.* https://www.bacb.com/services/o.php?page=100180

[CR8] Behavior Analyst Certification Board. (2023b). *BACB certificant registry.* https://www.bacb.com/services/o.php?page=101135

[CR9] Bosch, S., & Fuqua, R. W. (2001). Behavioral cusps: A model for selecting target behaviors. *Journal of Applied Behavior Analysis,**34*(1), 123–125. 10.1901/jaba.2001.34-123 11317984 10.1901/jaba.2001.34-123 PMC1284293

[CR10] Brodhead, M. T., Quigley, S. P., & Wilczynski, S. M. (2018). A call for discussion about scope of competence in behavior analysis. *Behavior Analysis in Practice,**11*, 424–435. 10.1007/s40617-018-00303-8 30538919 10.1007/s40617-018-00303-8 PMC6269378

[CR11] Capriotti, M. R., & Donaldson, J. M. (2022). “Why don’t behavior analysts do something?” Behavior analysts’ historical, present, and potential future actions on sexual and gender minority issues. *Journal of Applied Behavior Analysis,**55*(1), 19–39. 10.1002/jaba.884 34633066 10.1002/jaba.884

[CR12] Conine, D. E., Campau, S. C., & Petronelli, A. K. (2022). LGBTQ+ conversion therapy and applied behavior analysis: A call to action. *Journal of Applied Behavior Analysis,**55*(1), 6–18. 10.1002/jaba.876 34407211 10.1002/jaba.876

[CR13] Cooper, A. (Presenter). (2011). The sissy boy experiment [TV series episodes]. In: A. Cooper (Presenter), *Anderson Cooper 360*. CNN.

[CR14] Cox, D. J. (2020). A guide to establishing ethics committees in behavioral health settings. *Behavior Analysis in Practice,**13*, 939–949. 10.1007/s40617-020-00455-6 33269203 10.1007/s40617-020-00455-6 PMC7666231

[CR15] Davidson, M. (2017). Vaccines as a cause of autism–myths and controversies. *Dialogues in Clinical Neuroscience,**19*(4), 403–407. 10.31887/DCNS.2017.19.4/mdavidson 29398935 10.31887/DCNS.2017.19.4/mdavidson PMC5789217

[CR16] Deochand, N., Eldridge, R. R., & Peterson, S. M. (2020). Toward the development of a functional analysis risk assessment decision tool. *Behavior Analysis in Practice,**13*, 978–990. 10.1007/s40617-020-00433-y 33269209 10.1007/s40617-020-00433-y PMC7666254

[CR17] DeStefano, F., & Shimabukuro, T. T. (2019). The MMR vaccine and autism. *Annual Review of Virology,**6*(1), 585–600. 10.1146/annurev-virology-092818-015515 30986133 10.1146/annurev-virology-092818-015515 PMC6768751

[CR18] Digdon, N. (2020). The Little Albert controversy: Intuition, confirmation bias, and logic. *History of Psychology,**23*, 122–131. 10.1037/hop0000055 28125246 10.1037/hop0000055

[CR19] Digdon, N., Powell, R. A., & Harris, B. (2014). Little Albert’s alleged neurological impairment: Watson, Rayner, and historical revision. *History of Psychology,**17*(4), 312–324. 10.1037/a0037325 25068585 10.1037/a0037325

[CR20] Editors of The Lancet. (2010). Retraction–Ileal-lympoid-nodular hyperplasia, non-specific colitis, and pervasive developmental disorder in children. *The Lancet,**375*(9713), 445. 10.1016/S0140-6736(10)60175-4 10.1016/S0140-6736(10)60175-4 20137807

[CR21] Flowers, J., & Dawes, J. (2023). Dignity and respect: Why therapeutic assent matters. *Behavior Analysis in Practice*. 10.1007/s40617-023-00772-6 10.1007/s40617-023-00772-6 PMC10700258 38076752

[CR22] Frilund, A. J., Beck, H. P., Goldie, W. D., & Irons, G. (2020). The case for Douglas Merritte: Should we bury what is alive and well? *History of Psychology,**23*(2), 132–148. 10.1037/hop0000142 32378913 10.1037/hop0000142

[CR23] Goode, D., Hill, D. B., Reiss, J., & Bronston, W. (2013). *History and sociology of the Willowbrook State School*. American Association on Intellectual and Developmental Disabilities.

[CR24] Greely, H. T., & Cho, M. K. (2013). The Henrietta Lacks legacy grows. *EMBO reports,**14*(10), 849. 10.1038/embor.2013.148 24030280 10.1038/embor.2013.148 PMC3807222

[CR25] Harris, B. (1979). Whatever happened to Little Albert? *American Psychologist,**34*(2), 151–160. 10.1037/0003-066X.34.2.151

[CR26] Harris, B. (2020). Journals, referees, and gatekeepers in the dispute over Little Albert, 2009–2014. *History of Psychology,**23*, 103–121. 10.1037/hop0000087 32378912 10.1037/hop0000087

[CR27] Horton, R. (2004). A statement by the editors of *The Lancet*. *The Lancet,**363*(9411), 820–821. 10.1016/S0140-6736(04)15699-7 10.1016/S0140-6736(04)15699-7 15022645

[CR28] Johnson, A. H. (2022). The weight of harm: A response to “Editor’s Note: Societal changes and expression of concern about Rekers and Lovaas’ (1974) Behavioral treatment of deviant sex-role behaviors in a male child”. *Behavior Analysis in Practice,**15*(3), 971–979. 10.1007/s40617-022-00683-y 36457832 10.1007/s40617-022-00683-y PMC9582059

[CR29] Jones, S. H., St. Peter, C. C., & Ruckle, M. M. (2020). Reporting of demographic variables in the Journal of Applied Behavior Analysis. *Journal of Applied Behavior Analysis,**53*(3), 1304–1315. 10.1002/jaba.722 32383188 10.1002/jaba.722

[CR30] Joyce, N. R., & Rankin, T. J. (2010). The lessons of the development of the first APA ethics code: Blending science, practice, and politics. *Ethics & Behavior,**20*(6), 466–481. 10.1080/10508422.2010.521448

[CR31] Kelly, M. P., & Trifyllis, I. (2022). Developing national occupational standards for behavior analysis. *European Journal of Behavior Analysis,**23*(2), 196–214. 10.1080/15021149.2022.2137654

[CR32] Henrietta Lacks: Science must right a historical wrong [Editorial]. (2020). *Nature, 585*(7823), 7. 10.1038/d41586-020-02494-z 10.1038/d41586-020-02494-z 32873976

[CR33] LeBlanc, L. A., Nosik, M. R., & Petursdottir, A. (2018). Establishing consumer protections for research in human service agencies. *Behavior Analysis in Practice,**11*, 445–455. 10.1007/s40617-018-0206-3 30538921 10.1007/s40617-018-0206-3 PMC6269374

[CR34] Li, A., Wallace, L., Ehrhardt, K. E., & Poling, A. (2017). Reporting participant characteristics in intervention articles published in five behavior-analytic journals, 2013–2015. *Behavior Analysis: Research and Practice,**17*(1), 84–91. 10.1037/bar0000071

[CR35] Mead Jasperse, S. C., Kelly, M. P., Ward, S., Fernand, J. K., Joslyn, P. R., & van Dijk, W. (2023). Consent and assent practices in behavior analytic research. *Behavior Analysis in Practice.*10.1007/s40617-023-00838-5 Advance online publication 10.1007/s40617-023-00838-5 PMC12508419 41080008

[CR36] Milgram, S. (1963). Behavioral study of obedience. *Journal of Abnormal & Social Psychology,**67*(4), 371–378. 10.1037/h0040525 10.1037/h0040525 14049516

[CR37] Milgram, S. (1974). *Obedience to authority*. Harper.

[CR38] Morris, C., Detrick, J. J., & Peterson, S. M. (2021). Participant assent in behavior analytic research: Considerations for participants with autism and developmental disabilities. *Journal of Applied Behavior Analysis,**54*(4), 1300–1316. 10.1002/jaba.859 34144631 10.1002/jaba.859

[CR39] National Commission for the Protection of Human Subjects in Biomedical & Behavioral Research. (1979). *The Belmont report: Ethical principles and guidelines for the protection of human subjects of research*. U.S. Department of Health and Human Services. https://www.hhs.gov/ohrp/regulations-and-policy/belmont-report/read-the-belmont-report/index.html

[CR40] National Research Act of 1974, (1974) 42 U.S.C. § 201 et seq. https://uscode.house.gov/view.xhtml?path=/prelim@title42/chapter6A/subchapter3&edition=prelim

[CR41] Nordyke, N. S., Baer, D. M., Etzel, B. C., & LeBlanc, J. M. (1977). Implications of the stereotyping and modification of sex role. *Journal of Applied Behavior Analysis,**10*(3), 553–557. 10.1901/jaba.1977.10-553 924924 10.1901/jaba.1977.10-553 PMC1311225

[CR42] Normand, M. P., & Donohue, H. E. (2023). Research ethics for behavior analysts in practice. *Behavior Analysis in Practice,**16*, 13–22. 10.1007/s40617-022-00698-5 37006418 10.1007/s40617-022-00698-5 PMC10050523

[CR43] Nuremberg Military Tribunals. (1949). *Trials of war criminals before the Nuremberg Military Tribunals under Control Council law No. 10* (Vol. 2). U.S. Government Printing Office. https://archive.org/details/TrialsOfWarCriminalsBeforeTheNurembergMilitaryTribunalsUnderControlCouncil

[CR44] Pritchett, M., Ala’i-Rosales, S., Cruz, A. R., & Cihon, T. M. (2021). Social justice is the spirit and aim of an applied science of human behavior: Moving from colonial to participatory research practices. *Behavior Analysis in Practice,**15*, 1074–1092. 10.1007/s40617-021-00591-7 34178290 10.1007/s40617-021-00591-7 PMC8218790

[CR45] Protection of Human Subjects (1991), 45 C.F.R. Part 46. https://www.ecfr.gov/on/2018-07-19/title-45/subtitle-A/subchapter-A/part-46

[CR46] Qualified Applied Behavior Analysis Credentialing Board. (2021). *QABA ethical code of conduct*. https://qababoard.com/wp-content/uploads/Code-of-Ethics-03-25-21.pdf

[CR47] Rajaraman, A., Hanley, G. P., Gover, H. C., Staubitz, J. E., Simcoe, K. M., & Metras, R. (2022). Minimizing escalation by treating dangerous problem behavior within an enhanced choice model. *Behavior Analysis in Practice,**15*, 219–242. 10.1007/s40617-020-00548-2 35340377 10.1007/s40617-020-00548-2 PMC8854458

[CR48] Rekers, G. A., & Lovaas, O. I. (1974). Behavioral treatment of deviant sex-role behaviors in a male child. *Journal of Applied Behavior Analysis,**7*(2), 1973–190. 10.1901/jaba.1974.7-173 10.1901/jaba.1974.7-173 PMC1311956 4436165

[CR49] Rekers, G. A. (1972). *Pathological sex-role development in boys; Behavioral treatment and assessment* (Publication No. 7233978) [Doctoral dissertation, University of California, Los Angeles]. ProQuest Dissertations & Theses Global.

[CR50] Reynolds, G. (2006). The stuttering doctor's "Monster Study". In R. Goldfarb (Ed.), *Ethics: A case study from fluency*, Plural.

[CR51] Rice, T. W. (2008). The historical, ethical, and legal background of human-subjects research. *Respiratory Care,**53*(10), 1325–1329. 18811995

[CR52] Skinner, B. F. (1948). *Walden two*. Hackett.

[CR53] Society for the Experimental Analysis of Behavior, & LeBlanc, L. A. (2020). Editor’s Note: Societal changes and expression of concern about Rekers and Lovaas’ (1974) Behavioral treatment of deviant sex-role behaviors in a male child. *Journal of Applied Behavior Analysis,**53*(4), 1830–1836. 10.1002/jaba.768

[CR54] Taylor, L. E., Swerdfeger, A. L., & Eslick, G. D. (2014). Vaccines are not associated with autism: An evidence-based meta-analysis of case-control and cohort studies. *Vaccine,**32*(29), 3623–3629. 10.1016/j.vaccine.2014.04.085 24814559 10.1016/j.vaccine.2014.04.085

[CR55] Thompson, T. (2012). Joseph V. Brady: Synthesis reunites what analysis has divided. *The Behavior Analyst,**35*(2), 197–208. 10.1007/BF03392278 23450040 10.1007/BF03392278 PMC3501422

[CR56] Tobin, M. J. (2022). Fiftieth anniversary of uncovering the Tuskegee syphilis study. *American Journal of Respiratory & Critical Care Medicine,**205*(10), 1145–1158. 10.1164/rccm.202201-0136SO 35500908 10.1164/rccm.202201-0136SO PMC9872801

[CR57] Tolich, M. (2014). What can Milgram and Zimbardo teach ethics committees and qualitative researchers about minimizing harm? *Research Ethics, 10*(2), 86–96. 10.1177/1747016114523771

[CR58] U.S. Holocaust Memorial Museum. (2006). Holocaust encyclopedia: Nazi medical experiments. https://encyclopedia.ushmm.org/content/en/article/nazi-medical-experiments

[CR59] U.S. Holocaust Memorial Museum. (n.d.a). The doctors’ trial: The medical case of the subsequent Nuremberg proceedings. https://www.ushmm.org/information/exhibitions/online-exhibitions/special-focus/doctors-trial#:~:text=On%20December%209%2C%201946%2C%20an,crimes%20and%20crimes%20against%20humanity

[CR60] U.S. Holocaust Memorial Museum. (n.d.b). *Nuremberg code.* https://www.ushmm.org/information/exhibitions/online-exhibitions/special-focus/doctors-trial/nuremberg-code

[CR61] Wakefield, A. J., Murch, S. H., Anthony, A., Linnell, J., Casson, D. M., Malik, M., Berelowitz, M., Dhillon, A. P., Thompson, M. A., Harvey, P., Valentine, A., Davies, S. E., & Walker-Smith, J. A. (1998). Ileal-lymphoid-nodular hyperplasia, non-specific colitis, and pervasive developmental disorder in children. *The Lancet,**351*(9103), 637–641. 10.1016/S0140-6736(97)11096-0 10.1016/s0140-6736(97)11096-0 9500320

[CR62] Watson, J. B., & Rayner, R. (1920). Conditioned emotional reactions. *Journal of Experimental Psychology,**3*, 1–14. 10.1037/h0069608

[CR63] Weindling, P. (2022). From the Nuremberg “doctor’s trial” to the “Nuremberg code”. In S. Gallin & I. Bedzow (Eds.), *Bioethics and the Holocaust: A comprehensive study in how the Holocaust continues to shape the ethics of health, medicine and human rights. *Springer. 10.1007/978-3-031-01987-6_12

[CR64] Weindling, P., von Villiez, A., Loewenau, A., & Farron, N. (2016). The victims of unethical human experiments and coerced research under National Socialism. *Endeavour,**40*(1), 1–6. 10.1016/j.endeavour.2015.10.005 26749461 10.1016/j.endeavour.2015.10.005 PMC4822534

[CR65] West, C. (2008). The history of APS. *APS Observer, 21*(7). https://www.psychologicalscience.org/observer/the-history-of-aps

[CR66] Winkler, R. C. (1977). What types of sex-role behavior should behavior modifiers promote? *Journal of Applied Behavior Analysis,**10*(3), 549–552. 10.1901/jaba.1977.10-549 924923 10.1901/jaba.1977.10-549 PMC1311224

[CR67] World Medical Association. (2013). World Medical Association Declaration of Helsinki ethical principles for medical research involving human subjects. *JAMA Journal of the American Medical Association,**310*(20), 2191–2194. 10.1001/jama.2013.281053 24141714 10.1001/jama.2013.281053

[CR68] World Medical Association. (2017). World Medical Association Declaration of Geneva. https://www.wma.net/policies-post/wma-declaration-of-geneva/

[CR69] World Medical Association. (2022). World Medical Association International Code of Medical Ethics. https://www.wma.net/policies-post/wma-international-code-of-medical-ethics/

[CR70] World Medical Association. (1964). World Medical Association Declaration of Helsinki—ethical principles for medical research involving human subjects. https://www.wma.net/wp-content/uploads/2018/07/DoH-Jun1964.pdf 19886379

